# Editorial: Adolescent sexual and reproductive health challenges in low-income settings

**DOI:** 10.3389/fpubh.2023.1287625

**Published:** 2023-09-22

**Authors:** Nazmul Alam, Lisa Merry, Joyce L. Browne, Quamrun Nahar

**Affiliations:** ^1^Asian University for Women, Chittagong, Bangladesh; ^2^University of Montreal, Montreal, QC, Canada; ^3^University Medical Center Utrecht, Utrecht, Netherlands; ^4^International Centre for Diarrhoeal Disease Research (icddr, b), Dhaka, Bangladesh

**Keywords:** adolescent, sexual and reproductive health, low and middle income countries, gender equality, comprehensive sexuality education (CSE)

Adolescents and young adults (10–24 years old) represent a substantial proportion of the world's population and the majority live in low and middle-income countries (LMICs). Significant global attention has been given to improve Sexual and Reproductive Health Rights (SRHR) of adolescents and young adults, however, despite progress, the gains have been inequitable across countries largely due to harmful social norms, toxic gender stereotypes, and acute power imbalances in many LMICs, especially for females. In LMICs, poor infrastructure, poverty, and high levels of gender-based violence, have significantly impeded women and adolescent girls' access to SRH services. Moreover, a large number of adolescents and young adults, especially those living in conflict and temporary settlement centers, are at high risk of sexual violence, genital cutting, HIV and other sexually transmitted infections (STIs), unintended pregnancies, unsafe abortions, and maternal death. In our Research Topic for Frontiers of Public Health, our objective was to contribute to the advancement of SRHR among adolescents and young adults living in LMICs. As such, we solicited articles that addressed one or more of the following topics: child marriage, contraception, unwanted pregnancy, unsafe abortion, STI/HIV/AIDS including HPV vaccine coverages, gender-based violence, sex trafficking, and exploitation, poor menstrual hygiene practices, menstrual problems, and other gynecological illnesses, SRHR of adolescents in refugee settlements, adolescent-friendly health services, adolescent SRH policies, and governance.

Within this Research Topic, a number of manuscripts were published. Two focused on adolescents in Bangladesh and analyzed data from a recent national adolescent health and wellbeing survey; the first article, entitled “*Factors that provide protection against intimate partner physical violence among married adolescents in Bangladesh*,” found that 16% of married adolescent girls experienced intimate partners physical violence (IPPV) (Rahman et al.). Results showed that IPPV risk was lower when living with parents-in-law or parents and when girls were married to men aged 21 or older; IPPV risk was higher among girls with a longer duration of marriage and no living children. The second article, entitled “*What shapes attitudes on gender roles among adolescents in Bangladesh*,” showed that among unmarried adolescents, girls were significantly more likely compared to boys to hold egalitarian views regarding gender roles (58 vs. 19%) (Streatfield et al.). Egalitarian views were significantly higher among both girls and boys if they were members of social organizations, participated in adolescent programs and had completed at least grade 10. Taken together the two papers highlight the importance of education, policy (minimum age for marriage), and the implementation of community level interventions toward advancing gender-equity and protecting young women from IPPV, and its associated adverse health outcomes, in Bangladesh.

The topic of contraception and family planning, important for the prevention of pregnancy and averting the health and social consequences that are associated with unwanted pregnancy, were addressed in two papers. The article “*Social-psychological determinants of hormonal contraceptive use intentions among adolescent girls in the Bono East Region of Ghana*,” highlights how positive attitudes regarding hormonal contraceptives, and self-efficacy to access and use hormonal contraceptives, are positively correlated with hormonal contraceptive use intention, thus underscoring the importance of comprehensive sex education, including enhancing adolescents' skills and sense of competence to access and use contraceptives (Boamah-Kaali et al.). Similar conclusions were also drawn in the paper: “*What are the sources of contraceptives for married and unmarried adolescents: Health services or friends? Analysis of 59 low- and middle-income countries*” (Hellwig and Barros). Using national health survey data from 59 LMICs, it was shown that in almost half of the countries, the use of modern contraceptives is lower among sexually active adolescents when compared to sexually active adult women. Results also showed, that in a number of countries, adolescents are less likely to access contraceptives in the public sector, especially unmarried girls. In all regions, unmarried girls, compared to their married counterparts, were more likely to access contraceptives from friends and family. Another article, which addressed the use of reproductive more broadly, further underscored the importance of sex education, and also the necessity for services to be easily accessible; in “*Reproductive health service use and associated factors among youths in Becho district, southwest Ethiopia*,” the researchers found that the use of reproductive health services was relatively low (44% utilized services) among youth and that knowledge of services, discussions with parents about reproductive health, as well as close proximity of a health facility, were key predictors of reproductive health service use (Waga et al.).

Lastly, implementation and sustainability of sexual and reproductive health services, was a topic in two papers. The article: “*Planning, implementation, and sustaining high coverage of human papillomavirus (HPV) vaccination programs: What works in the context of low-resource countries?*,” provides a comprehensive overview of challenges and facilitators to HPV vaccination program implementation in LMICs (Waheed et al.). Results showed that data availability, management and reporting and supply chain difficulties are significant issues, while involvement of multi-sectoral key stakeholders, careful planning and coordination, and communication and training at all levels (district administrators, healthcare staff, teachers, and support personnel), are vital toward ensuring successful and sustained implementation of HPV programs. In their paper, entitled: “*Accelerated institutionalization of an adolescent sexual and reproductive health (ASRH) intervention in Tanzania: Findings from a mixed-methods evaluation*,” the authors describe the institutionalization and scale-up of a contraceptive-delivery intervention into government healthcare institutions across Tanzania; the intervention, which includes in-clinic care and a community-outreach component, aims to educate, but also to empower girls by focusing on life planning and skills acquisition (Cutherell et al.). Commitment and early, active involvement of key stakeholders, including government officials and healthcare providers, and ongoing engagement and training, were also identified in this study as important facilitating factors for successful implementation of the intervention. Integrating the intervention within existing structures, was also shown to facilitate the institutionalization process.

In sum, the research reported in this Research Topic highlights the critical role that governments in LMICs must play to ensure SRH services are available, easily accessible and responsive to the needs of adolescents and youth; these services should provide education, enhance self-efficacy and improve skills with regards to accessing and using services and care, but also equally important, is the empowerment of girls. Policies are also crucial; they should promote young people to study longer and to delay marriage until a later age. The studies also emphasize the importance of comprehensive sexuality education (CSE) within school to improve knowledge, but also to eventually shift cultural and social norms that contribute to poor SRH outcomes, especially for girls and young women. Overall, the authors conclude that more evidence and continued work by researchers, care-providers and policy makers, are still needed to improve gender equality and the SRH of adolescents and youth in LMICs. The work reported in this Research Topic offers a point of departure for those looking to contribute and advance the SRH field. We would like to express our sincere gratitude to the authors, reviewers and editors of Frontiers in Public Health, for their contributions to this Research Topic.

## Author contributions

NA: Writing—original draft. LM: Writing—review and editing. JB: Writing—review and editing. QN: Writing—review and editing.

